# A case of Meckel’s diverticulum misdiagnosed as gastrointestinal stromal tumor: case report and literature review

**DOI:** 10.3389/fmed.2025.1672019

**Published:** 2025-12-19

**Authors:** Binbin Wang, Shuxin Li, Gengchen Huang, Zhijun Tang, Zihao Ye, Miao Wang, Wei Wei

**Affiliations:** 1 Department of Urology II, The First Hospital of Jilin University, Changchun, China; 2 Department of Gastrointestinal and Colorectal Surgery, The First Hospital of Jilin University, Changchun, China

**Keywords:** Meckel’s diverticulum, ectopic gastric mucosa, gastrointestinal stromal tumor, laparoscopic surgery, case report

## Abstract

Meckel’s diverticulum (MD) is a common congenital gastrointestinal malformation often containing ectopic gastric mucosa. It is prone to ulceration and painless lower gastrointestinal bleeding, predominantly affecting children and adolescents. Gastrointestinal stromal tumors (GISTs), which arise from Cajal interstitial cells, are predominantly mesenchymal tumors that occur predominantly in middle-aged and elderly individuals. Both conditions may occur in the small intestine, presenting gastrointestinal bleeding and exhibiting overlapping imaging features, which pose challenges for clinical differentiation. This report describes a 17-years-old female patient admitted to the First Hospital of Jilin University with intermittent melena, abdominal pain, and anemia. Small bowel CT imaging (CTE) revealed a nodular lesion measuring approximately 0.9 cm × 1.8 cm within the ileal lumen. The lesion exhibited a broad base attached to the intestinal wall and showed marked homogeneous enhancement, strongly suggesting a GIST. Previous gastrointestinal endoscopy had only indicated chronic gastritis and colitis. The patient underwent laparoscopic segmental resection of the ileal mass. Postoperative pathology confirmed an MD with fundic gland-type ectopia. This case highlights the diagnostic challenges of complex MD and underscores the critical role of histopathology. It thereby provides diagnostic and surgical guidance for MD cases that mimic GIST on imaging, thereby reducing misdiagnosis.

## 1 Introduction

Meckel’s diverticulum (MD) is a common congenital gastrointestinal malformation resulting from incomplete regression of the embryonic vitelline duct (1). Its clinical features are often summarized by the “Rule of 2s”: an incidence of approximately 2%, commonly located on the para mesenteric margin within the proximal 2 feet (approximately 61 cm) of the ileocecal valve, measuring about 2 inches (approximately 5 cm) in length, with a male predominance (approximately 2:1), and symptoms typically presenting before age 2 (1, 2). As a true diverticulum, MD comprises the entire wall thickness of the small intestine, with the inner lining potentially containing ectopic tissue; among these, gastric mucosal ectopia is the most common, observed in approximately half of MD cases (3). This ectopic tissue is closely associated with clinical symptoms, with a detection rate as high as 80% in symptomatic patients. Furthermore, all bleeding patients exhibit gastric mucosal ectopia in the fundus and body regions (4, 5). This occurs because gastric acid secreted by the ectopic mucosa erodes adjacent ileal mucosa, causing ulcers and gastrointestinal bleeding, significantly increasing complication risks (5). However, most MD patients remain asymptomatic throughout life, with only a minority presenting due to complications (6). Preoperative diagnosis rates in symptomatic patients are less than 10% (7). Therefore, young patients with negative upper and lower gastrointestinal endoscopy findings but bleeding should be evaluated for the possibility of MD (8).

Gastrointestinal stromal tumors (GISTs) are rare tumors accounting for 1%–2% of gastrointestinal malignancies, yet they represent the most common mesenchymal tumors of the digestive tract, comprising approximately 80% of such tumors (8). They predominantly affect adults and older individuals, with rare cases in children (9). GISTs can involve the entire digestive tract, primarily affecting the stomach and small intestine, while involvement of the colon and esophagus is uncommon (10). GISTs often present with minimal symptoms and are frequently incidentally detected on imaging studies. Some may manifest as abdominal pain or gastrointestinal bleeding (11). They exhibit diverse growth patterns and possess a rich blood supply, often showing marked enhancement during the arterial phase of CT scans (12). Notably, GIST is extremely rare in adolescents, while MD is a common cause of lower gastrointestinal bleeding in this population (13, 14). Hemorrhage, MD’s most frequent complication in adolescents, accounts for 40%–50% of symptomatic cases and almost only occurs with heterotopic gastric mucosa (14). However, overlapping CT features between small MD with ectopic gastric mucosa and small GIST often lead to preoperative misdiagnosis. This report describes a 17-years-old female patient with lower gastrointestinal bleeding secondary to a complex MD. Preoperative small bowel CT suggested a possible GIST, but postoperative pathology confirmed MD with fundic gland ectopia.

## 2 Case presentation

**FIGURE 1:**
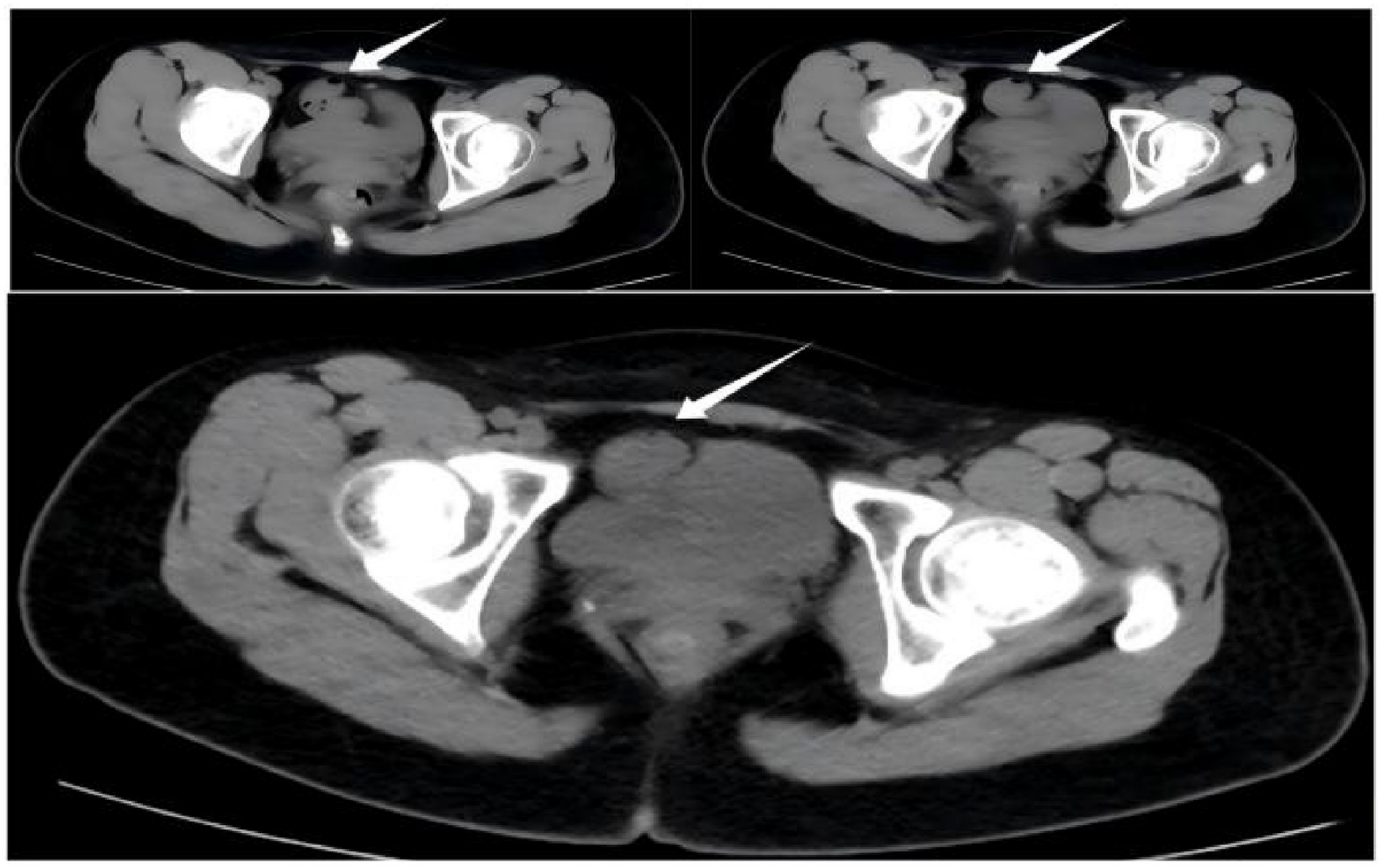
Non-contrast multi-slice CT images of the patient’s entire abdomen. A slightly increased density within the small bowel lumen in the pelvic region is visible, indicated by the white arrow.

**FIGURE 2:**
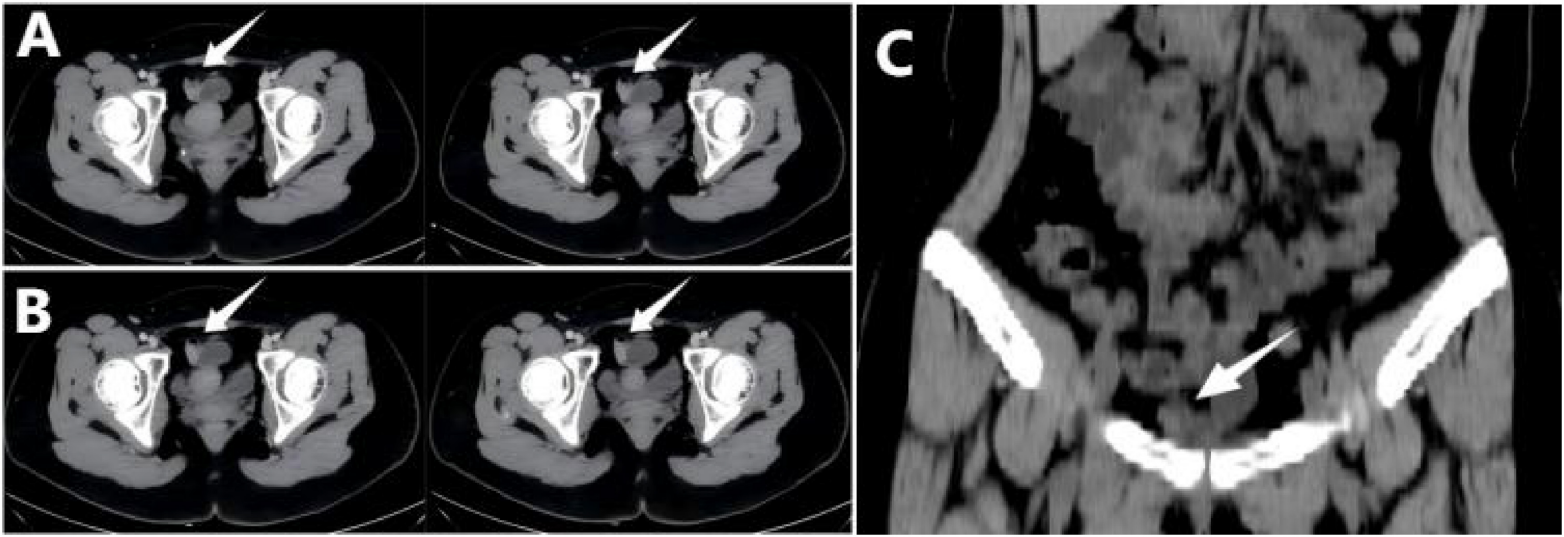
Multi-slice CT imaging of the small intestine. **(A,B)** Axial views show a single nodular hyperdense lesion with relatively well-defined borders, indicated by white arrows. **(C)** The coronal view demonstrates the corresponding hyperdense lesion, indicated by a white arrow.

A 17-years-old female patient presented to the First Hospital of Jilin University with intermittent melena lasting over 10 days. Upon admission, vital signs were stable. Ten days prior, she developed intermittent epigastric pain without apparent cause, followed by passage of loose, tarry black stools. Key laboratory findings were as follows: Complete blood count (CBC) showed red blood cells (RBC) 2.70 × 10^12^/L (reference range, RR 4.1–5.3 × 10^12^/L), hemoglobin (HGB) 71 g/L (RR 114–154 g/L), Hematocrit (HCT) 0.231 L/L (RR 0.36–0.47 L/L), Mean Corpuscular Hemoglobin Concentration (MCHC) 307 g/L (RR 310–355 g/L), Platelet count (PLT) 343 × 10^9^/L (RR 150–407 × 10^9^/L); high-sensitivity C-reactive protein 1.22 mg/L (RR 0–1.0 mg/L); D-dimer 0.87 mg/L FEU (RR 0.00–0.50 mg/L FEU). No significant abnormalities noted in the coagulation panel. Previous endoscopic studies at a local hospital (1 year prior) revealed chronic non-atrophic gastritis on painless electronic gastroscopy and proctitis on painless electronic colonoscopy. Whole-abdominal multi-slice CT scan showed slightly increased density within the small bowel lumen in the pelvic region (Figure 1). One week before admission, painless electronic gastroscopy at this hospital demonstrated chronic non-atrophic gastritis; Painless colonoscopy revealed chronic colitis; small bowel multi-detector CT imaging showed a nodular hyperdense lesion visible within the intestinal lumen extending from the small bowel to the level of both hip joints, attached to the intestinal wall with a broad base and protruding into the lumen, measuring approximately 0.9 cm × 1.8 cm, with a CT value of roughly 36 Hounsfield units (HU). It demonstrated marked and uniform enhancement on contrast-enhanced scanning with relatively straightforward margins, highly suggestive of a stromal tumor (Figure 2). Based on the above findings, laparoscopic segmental resection of the ileal mass was planned to determine the nature of the lesion and achieve precise excision.

**FIGURE 3:**
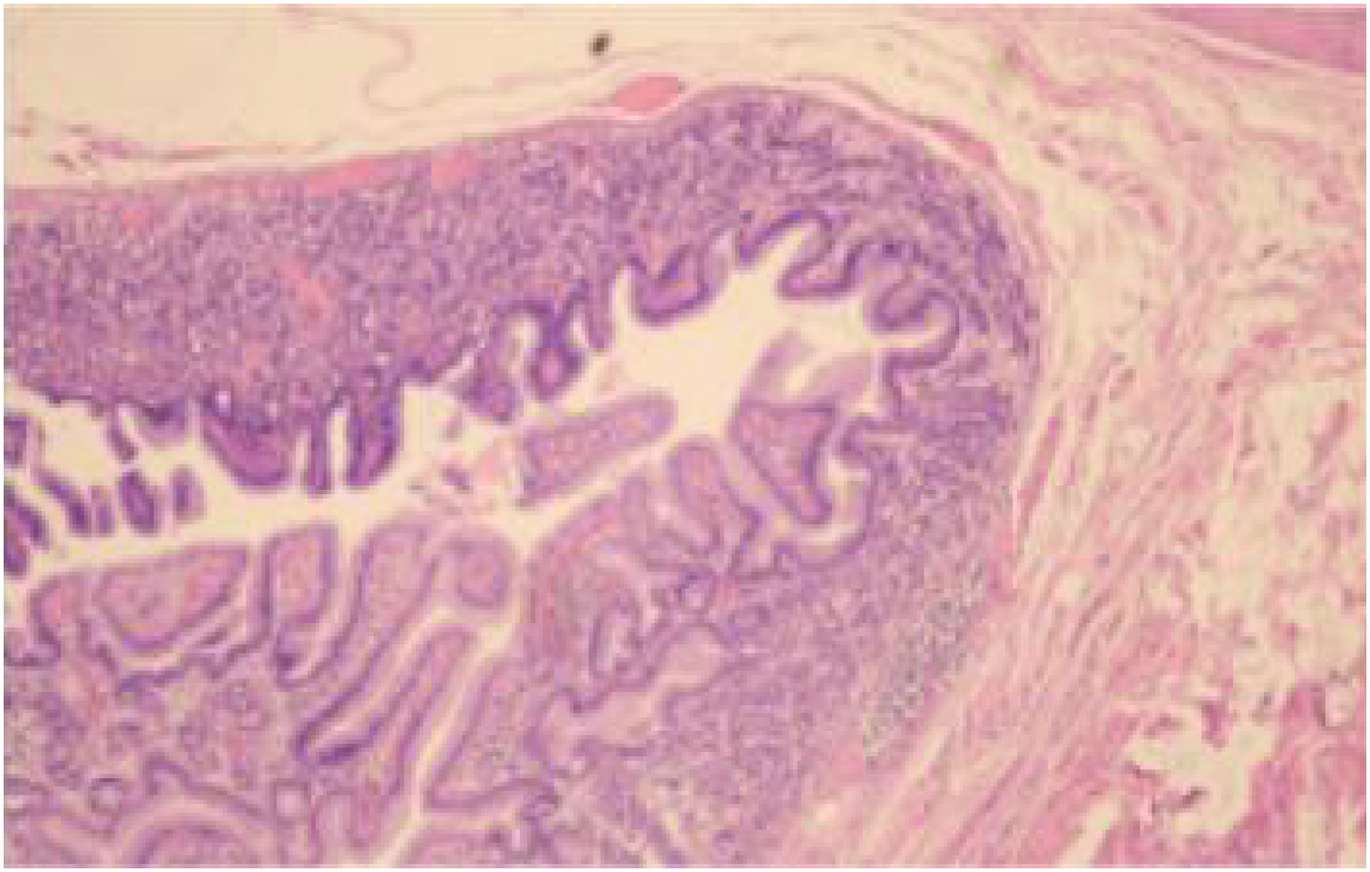
Postoperative pathological diagnosis report of the patient. Light microscopy image (HE staining, 100× magnification). The pathological diagnosis is a small bowel diverticulum with fundic gland ectopia.

**Treatment course:** The patient underwent laparoscopic segmental resection of an ileal mass. During surgery, a lesion was identified in the small intestine approximately 60 cm from the ileocecal junction. A linear mechanical stapler was used to perform segmental resection, with the specimen sent for pathological examination. No abdominal drainage tubes were placed intraoperatively, and no surgical complications occurred. The postoperative pathological diagnosis was small bowel diverticulum with fundic gland ectopia (Figure 3). The patient recovered well postoperatively and was discharged on the third postoperative day. During a telephone follow-up 6 months postoperatively, the patient reported good recovery with no abdominal pain, melena, or other discomfort.

## 3 Discussion

Meckel’s diverticulum is the most common congenital anomaly of the small intestine. Most patients remain asymptomatic throughout their lives, with only 4%–9% requiring clinical intervention due to complications such as intestinal obstruction, bleeding, or diverticulitis (15). As a true diverticulum formed by incomplete closure of the yolk sac during the embryonic period (gestational weeks 5–7), its typical anatomical location is approximately 60 cm proximal to the ileocecal valve on the contralateral margin of the mesentery (16). However, mesenteric MD variants have also been reported (2). This variant, due to its atypical location, is more prone to confusion with neoplastic lesions such as GIST, increasing the risk of preoperative misdiagnosis. In stark contrast, GIST, the most common mesenchymal tumor of the gastrointestinal tract, arises from Cajal interstitial cells and characteristically harbors KIT or PDGFRA mutations (17). They predominantly affect middle-aged and elderly individuals, with rare cases in adolescents, whereas MD primarily affects children and adolescents. This age discrepancy serves as a crucial preliminary diagnostic clue.

Overlapping imaging features constitute the primary cause of misdiagnosis between MD and GIST, as both may present as focal, protruding, mass-like lesions within the intestinal wall (18, 19). GISTs exhibit wide size variability, ranging from less than 1 cm to over 20 cm in diameter (20). Smaller GISTs (<5 cm) typically display well-defined margins and homogeneous enhancement after contrast administration (21), which is also the characteristic CT feature of small (<5 cm) small bowel GISTs (22). However, MDs containing ectopic gastric mucosa may also exhibit similar homogeneous enhancement due to their rich mucosal vascularity. When MDs develop secondary inflammation or edema, the imaging features of intestinal wall thickening and increased surrounding soft tissue density can further confound the local mass appearance of GISTs (19). Particularly in this case, the small size (0.9 cm × 1.8 cm) and homogeneous enhancement of the lesion increased the likelihood of CT misdiagnosis. However, key distinctions remain: typical MDs often evolve into thin-walled, smooth-surfaced cystic structures communicating with the intestinal lumen (containing fluid or gas) as the lesion progresses (23), whereas larger GISTs typically present as solid masses, frequently accompanied by central necrosis or ulceration, and demonstrate heterogeneous enhancement post-contrast (24). Considering the patient’s age (17 years old–adolescent GIST is extremely rare) and lesion location (close to the typical metastatic distribution area), integrating age, anatomical location, and imaging features for comprehensive preoperative analysis can reduce the probability of misdiagnosis.

This case further highlights the value of specialized examinations in differentiating MD from GIST. Among adolescents with unexplained gastrointestinal bleeding, 50% are associated with MD (25). Technetium-99m pertechnetate scanning, serving as the gold standard for detecting ectopic gastric mucosa, achieves a diagnostic accuracy of 90% in adolescent patients (26). Its typical imaging features include focal tracer accumulation in the right lower abdomen, synchronous gastric mucosal uptake in the early phase, and persistent delayed-phase enhancement (27). They can precisely identify ectopic gastric mucosa of varying sizes and locations (28). However, false-positive results may occur due to physiological uterine uptake during menstruation in females (29). Capsule endoscopy allows direct visualization of active bleeding, intussusception, or the double-lumen sign, but its clinical application remains limited (30, 31). Therefore, for young bleeding patients with atypical CT findings, prioritizing the specialized examinations can effectively improve preoperative diagnostic accuracy and prevent misdiagnosis or inappropriate treatment. Furthermore, clinical management requires clear differentiation between asymptomatic MD and incidental MD: the former refers to MD without associated clinical symptoms such as bleeding, abdominal pain, or intestinal obstruction; the latter denotes explicitly MDs incidentally detected during abdominal surgery for other conditions like appendicitis or intestinal obstruction, or during gastrointestinal imaging studies such as ultrasound, CT, or small bowel contrast studies. Such MDs are predominantly categorized as asymptomatic (14, 32, 33). For symptomatic MD, surgical resection remains the only curative approach. Standard procedures include diverticulectomy, wedge resection, and segmental bowel resection. Laparoscopic surgery, with its lower complication rate, has become the clinical preference (34). Regarding treatment strategies for asymptomatic MD and incidental MD, controversy persists with no unified consensus. Clinical decisions require careful balancing of surgical risks against potential benefits from preventing complications (35, 36). Most experts advocate against routine resection, primarily because the overall complication rate of MD is low. The risks associated with routine surgery–such as infection, postoperative intestinal obstruction, and anastomotic leakage–often outweigh the potential benefits of prophylactic resection (14). However, some studies suggest that prophylactic resection may be considered for asymptomatic MD in pediatric patients to reduce the risk of long-term complications or malignant transformation (37). If a patient has undergone an exploratory laparotomy for another condition, incidental MD can be proactively resected during the same procedure to avoid future reoperation for complications (14). Therefore, MD management should adhere to patient-centered, individualized principles, incorporating factors such as age, presence of ectopic gastric mucosa, and surgical context to avoid a one-size-fits-all approach (38).

In this case, the patient’s prolonged intermittent abdominal pain and fatigue stemmed from chronic ulcer bleeding caused by ectopic gastric mucosa secreting gastric acid, manifesting as chronic anemia. Such early symptoms of slow blood loss are often subtle and easily overlooked clinically. Additionally, the discrepancy between preoperative CT findings and postoperative pathology underscores CT’s limitations in distinguishing MD from GIST. It is crucial to recognize that misdiagnosis between these two conditions can have severe consequences: misidentifying MD as GIST may lead to excessive surgical resection, while misidentifying GIST as MD may delay targeted therapy, potentially causing high-risk patients to miss the optimal treatment window (39, 40).

In summary, preoperative differentiation between MD and GIST requires integrating multidimensional evidence from age, location, and specialized imaging. Additionally, it is essential to clarify the definition distinctions between asymptomatic and incidentally detected MD, and to formulate individualized treatment strategies based on the latest clinical evidence. This case highlights that when adolescents present with small bowel masses and bleeding, comprehensive evaluation is imperative - imaging - specialized testing. It is also crucial to clarify the defining differences between asymptomatic and incidentally detected MDs, and to formulate individualized treatment strategies based on the latest clinical evidence. This case highlights that when encountering adolescents with small bowel masses accompanied by bleeding, clinicians should prioritize the reference value of age characteristics and anatomical location. When necessary, incorporating specialized testing can enhance diagnostic accuracy, which is significant for optimizing the diagnostic and therapeutic process and improving patient outcomes.

## 4 Conclusion

This case highlights the diagnostic challenge of distinguishing Meckel’s diverticulum from gastrointestinal stromal tumor in young patients, given overlapping imaging features. An accurate preoperative diagnosis relies on integrating key clinical clues, particularly patient age and lesion location. The selective use of targeted investigations is crucial to avoid misdiagnosis. Ultimately, a multidimensional approach is essential for guiding appropriate, individualized management.

## Data Availability

The original contributions presented in this study are included in this article/supplementary material, further inquiries can be directed to the corresponding author.
